# Physical activity among Norwegian adolescents- a multilevel analysis of how place of residence is associated with health behaviour: the Young-HUNT study

**DOI:** 10.1186/1475-9276-12-56

**Published:** 2013-07-24

**Authors:** Brit Logstein, Arild Blekesaune, Reidar Almås

**Affiliations:** 1 Centre for Rural Research (University Centre Dragvoll), Norway 7491, Norway; 2 Department of Sociology and Political Science, Norwegian University of Science and Technology (University Centre Dragvoll), Trondheim 7491, Norway

**Keywords:** Physical activity, Adolescents, Informal social capital, Area deprivation, The Young-HUNT Study

## Abstract

**Introduction:**

The purpose of this article is to investigate whether and to what degree participation in physical activity among adolescents is associated with area economic deprivation in the municipality where they live. In the study we took account of aggregated informal social capital at the municipality level and compositional effects due to spatial concentration of individual variables known to be associated with physical activity. These include informal social participation, participation in other cultural activities, and family affluence.

**Methods:**

The study was based on a secondary analysis of data from the Norwegian HUNT study and municipality characteristics from the Norwegian Social Science Data Service ‘Commune Database’ from 2006. The sample consisted of 8114 adolescents whose ages ranged from 13 to 19. The explanatory power of the independent variables on the dependent variable was assessed using a multilevel analysis in which individuals comprised the first level and were nested within the municipality level.

**Results:**

The average level of physical activity was not negatively associated with the level of area economic deprivation when we adjusted for informal social participation at the community level. Adjusting for area economic deprivation, we found that informal social participation at the community level was associated with a higher level of participation in physical activity at p< .01.

**Conclusion:**

For adolescents in a given municipality, informal social participation is more strongly associated with a higher level of physical activity than the degree of area economic deprivation. This finding supports our social capital hypothesis, which states that the amount of social capital is strengthening the individual’s ability to take part in physical activity.

## Introduction

Recent research supports the long-held opinion that physical activity has significant health benefits for youth. Participation in regular and varied physical activity is important to promote muscle strength and flexibility among young people [1] and in preventing obesity [2]. A favourable association has been found between physical activity and prevention of the metabolic syndrome [3]. It is also proposed that physical activity might enhance psychological well-being, and that a highly sedentary lifestyle is associated with inferior mental health among children and adolescents [4]. Even modest amounts of physical activity can result in impressive health benefits for school-aged children and youth [5]. However, in the western world, most young people are not physically active enough to achieve the beneficial health effects of physical activity in terms of present and future health status and well-being [6]. Strategies for the promotion of physical activity among adolescents should include identification of structural dimensions as well as societal, cultural, and personal factors that affect the development and maintenance of long-term increases in physical activity patterns [7,8]. A number of psychological factors are found to predict future participation in physical activity, such as self-motivation to exercise [9] and self-efficacy, understood as the individual’s confidence in certain activities [10]. However, physical activity among young people is a complex behaviour determined by many factors [11] and the decision to participate is not based on rational factors alone.

In recent decades, there has been increasing interest in how participation in physical activity varies according to place of residence. Piro et al. [12] found that people who live in certain parts of Oslo, Norway’s capital, participate less in physical activity than those who live in other parts of the city. In a number of studies, researchers have attempted to investigate whether area-related differences in health activities are due to the composition of the residents’ population, *the compositional properties*, or to features of place not captured by the individual properties, *the contextual characteristics*[13]. A compositional explanation of spatial-related differences in physical activity would be that areas include different social groups, and that these socioeconomic and cultural differences account for the observed variations in physical activity between locations. In these cases, the focus is on how the individual outcome variables, such as physical activity, are related to individual exposure variables, and on how these individual variables are clustered within and between geographical areas. In a contextual explanation, it is believed that natural or societal features of a specific area, not captured by the individual properties, are associated with individual outcome variables [14].

### Previous research concerning health-related activities among adolescents

As in the adult population, we find social inequalities in health behaviour such as physical activity among adolescents in the western world. Associations have been found between adolescents’ level of physical activity and parental education [15,16], occupational status of the parents [17], and family income [18]. These associations between socioeconomic status of the family and physical activity among adolescents are, however subject to debate [19]. It is believed that other social factors associated with peer groups and youth culture might cut across the indicators of socioeconomic status at the family level [20] or other characteristics of the family, such as family sense of belonging [21].

According to Morrow [22], the influence of parents on the lives of children and adolescents has been overstated in research, and the importance of social participation among young people is yet to be fully explored. Among adolescents, statistically significant relationships are found between being involved in different youth clubs or organizations and physical activity [21]. Among pre-adolescent children, physical activity is found to be positively associated with the amount of perceived social support an individual gets from friends [23]. A comprehensive review [11] demonstrated a lack of consistency for associations with social variables, even though some studies demonstrated that support from ‘significant others’ was consistently related to adolescents’ physical activity, while peer modelling of physical activity was unrelated.

The social structure surrounding an individual constitutes what is often referred to as social capital. Putnam [24] defines social capital as features of social organization, such as networks, norms, and trust. Such kind of social organizations facilitate coordination ant cooperation for mutual benefit, and the associated norms of reciprocity have values for those involved in the networks. In a certain social network, the norms within the social group might differ from other social networks. Closely knit peer groups without adult supervision may generate a high level of social capital that enables the individual member to reach the goal of the adolescents, but they might be opposite to the goals and norms of their parents [25,26]. In this article, we understand informal social participation with friends without adult supervision and characterized as more or less unstructured and spontaneous, as informal social capital, while formal social capital is social participation in networks and activities characterised as structured and in the supervision of their parents or others. The effect of formal and informal social capital on health behaviour among adolescents might thus differ, because the goals within social networks might not be the same [26]. Participation in physical activity in western societies is highly valued in the mainstream society because, among other reasons, of the known health benefits. According to Abel [27] and his theoretical review, culture-related factors such as normative beliefs, knowledge, and behaviour are positively associated with healthy behaviour. The reason is that cultural resources such as values and knowledge tend to structure preferences and choices, including physical activity habits.

Abel [27] draws on the work of Bourdieu, where cultural capital is defined as the repertoire of cultural signals. In his work on French society, Bourdieu [28] understood cultural capital as the degree of cultural confidence in which an individual is in possession of cultural competence and participates in activities that a majority of the people in the society regards as highly cultural. A somewhat similar study among Norwegian adolescents found a positive association between the number of books at home and the adolescent’s participation in physical activity, proposing a positive association between cultural capital and participation in physical activity [29]. That corresponds to the findings of Stempel [30], who claims that participating in sport actually operates as cultural capital, at least in the US adult population.

According to Bourdieu [31], our cultural interests and activities are not accidental. The individual’s social position in society is defined by his or her amount of economic, cultural, and social capital, and the relations between different forms of capital. In the case of sport, Bourdieu [31] widens the concept of cultural capital, asserting that sport is part of one person’s cultural capital and the whole range of sporting activities is regarded as a supply intended to meet a social demand. However, he also proposes that within a social class, there is both a dominating and dominated faction that differs in terms of participation in certain activities. Thus there might be social groups who prefer sporting activities to other cultural activities such as going to a concert or reading books, and vice versa.

At the community level, when we adjust for the individual characteristics of those living in a certain area, there might still be factors that are associated with adolescents’ physical activity. One of them is social capital at the community level. Societies with high levels of social capital are characterized by high levels of generalized trust in other people, as well as high levels of institutional trust and reciprocity among those living there [32]. It is believed that local communities high in social capital are better able to realize common values and support accepted community goals [25]. Coleman [33] understands social capital differently from Putnam [24]. According to Coleman (ibid.), social capital is generated in social networks and is thus a product of social networks, consequently contributing to the welfare of the community as a whole. According to Berkman and Kawachi [34], communities with an abundance of social capital may be better able to reinforce positive social norms for health behaviour, e.g. physical activity.

Another characteristic of the area of residence that might be related to the level of physical activity among the inhabitants is area economic deprivation. That is a collective measure of average socioeconomic positions of populations living within a particular area [34]. A study carried out in Oslo in Norway, found that adults living in the most deprived areas exercised less than those living in more affluent areas when adjustment is made for all individual socio-demographic variables [12]. Area economic deprivation was measured by using an index consisting of the following items: proportion of the population affected by social security benefits, proportion of unemployment, proportion receiving disability pension, proportion with no college or university education, and average taxable income in the area. In this study (ibid.), area economic deprivation was defined as the clustering of people with limited possibilities for choosing destination of residence: those who live there are subject to economic constraints preventing them from settling anywhere they want. An association between less physical activity and area economic deprivation was also reported by Sundquist et al. [35], who found that adults living in deprived areas of Sweden were more likely to be physically inactive than those living in affluent areas. In a review study carried out by Riva et al. [36], greater levels of area deprivation were also found to be associated with a greater likelihood of unhealthy dietary habits, smoking, overweight, and obesity.

It seems plausible that these associations also exist for Norwegian adolescents in Norway, even though research findings on this subject from other countries are mixed. According to Papayo et al. [37], physical activity was lower among boys from highly economically deprived areas than among boys from economically advantaged areas, while the opposite was found for girls. However, place of residence in terms of degree of area deprivation seems to affect adolescents in one way or another. Area deprivation, adjusted for individual variables, is found to affect behavioural problems such as drinking and smoking among young adolescents [38,39]. To explain how area deprivation might be associated with physical activity among the inhabitants, it is proposed that areas with a high degree of deprivation adding further disadvantage at the individual level and the effects of area deprivation might interact with difficulties at the individual level. However, it has proven difficult to demonstrate these processes empirically. Both in Sweden [40] and in Scotland [41], it was found that differences in health and health behaviour in deprived neighbourhoods were not explained by a lack of health-promoting resources. Anderson et al. [42] claim that low socioeconomic status of an area constitutes a health risk to its inhabitants because of its ecological concentration of poverty, unemployment, economic disinvestment, and social disorganization. According to Macintyre [13], both material or infrastructural resources and factors related to collective social functioning and practices might be relevant for local areas to be health promoting or health damaging beyond the effect of individual factors.

In Norway, it is on the public agenda that people should have the same access to health-promoting resources in their neighbourhood regardless of where they live. According to the Ministry of Health and Care Services in Norway and their Report to the Storting (2011–2015), it is thus essential that place of residence, neighbourhood, and the local community are included in the planning and implementation of actions to promote health and prevent diseases in the population. An issue that arises therefore is whether the level of physical activity among Norwegian adolescents varies by the place of residence despite these national initiatives in Norway, which are driven by the political target of reducing and preventing the development of health inequalities in the Norwegian population. The aim of this study is therefore to analyse the degree to which area deprivation at the level of the municipality where adolescents live is associated with physical activity, taking into account the magnitude of informal social participation among the adolescents at both the individual and the municipality level. Our main hypothesis, which may be called the social capital hypothesis, is that the size and composition of social capital will increase the individual's ability to participate in physical activity.

## Methods

Two data sets were combined, namely population-based individual data from the HUNT Study (“Helseundersøkelsen i Nord-Trøndelag”) and municipality characteristics from the Norwegian Social Science Data Service ‘Commune Database’. In this study, we used the youth sample in the HUNT3 Survey, Young-HUNT 3 Survey, carried out in 2006–2008. The HUNT study is a total population-based health study, and in the Young-HUNT3Survey, all residents in the county of Nord-Trøndelag in Norway aged 13 to 19 were included. The study county, Nord-Trøndelag, is one of 19 counties in Norway, situated in the middle of the country. The level of education and the level of income in the adult population are somewhat lower than the national average, but this county is in general considered representative of the whole of Norway (Krokstad, 2004). All adolescents in the appropriate age group were invited (10 464) to participate in the study, and the response rate was 78%. The final sample in this study was 8114 and the mean age was about 16 years (SD 1.72); gender was equally divided. The study was approved by the Regional Committees for Medical Research Ethics (REK) in Norway.

### Dependent variable

The dependent variable was one item in the health survey: “Outside school, how many hours a week do you participate in sport or exercise in your free time so much that you get out of breath or sweat?” The response alternatives were: “None”, “About ½ hour”, “About 1 hour”, “About 2 to 3 hours”, “About 4 to 6 hours” and “7 hours or more”. The response alternatives were interpolated from a five-point Likert scale to a scale ranging from 0 to 7, indicating the approximate number of hours of physical activity. On this variable there were 182 missing observations, and these were replaced by a mean value. When the missing observations were replaced by the mean value, the variance on that variable did not change substantively. SD = 2.37 when excluding cases and SD = 2.35 when replaced by the mean value. The mean value on the variable remained the same; 3.72.

### Exposure variable, municipality/contextual level

Characteristics of the 24 municipalities in Nord-Trøndelag County in 2006–2008 were found in the ‘Commune Database’, provided by Statistics Norway (http://www.kommune-databasen.no/). To assess the degree of deprivation at the municipality level, we used the following five deprivation indicators at the municipality level from 2006: The prevalence of low educational level, the prevalence of low mean income, the prevalence of disability pension, the prevalence of unemployment, and the relative amount of population decline. This way of operationalizing area deprivation also coincides with the approach used by Pabayo et al. [37] and Krokstad et al. [14]. A higher deprivation score reflects greater economic deprivation of the area in which the adolescent lives. Before the calculation of a sum score, all five items were given their standardized z-scores to ensure a standardized relative importance of each individual item in the index calculation. Cronbach’s alpha was 0.72. The deprivation index was linked to the individual HUNT data by the municipality number for each of the 24 municipalities in Nord-Trøndelag County.

### Control variables, individual level, and municipality level/contextual level

Cultural participation at the individual level was measured by six items that asked the respondents to report how often during the last week they had actively participated in or been a part of the audience in the following activities: theatre/show, film/photo, concert, choir, library, and cinema. The choice of which variables of cultural participation to include was based on both statistical and theoretical considerations. A number of leisure activities were included in a Principal Component Analysis (PCA) to narrow a high number of items, and these six items loaded relatively strongly on one component. The sum scores were calculated by adding together all individual values and dividing by the number of items (six). The numbers of missing observations on each individual variable ranked between 460 and 490, and these were replaced by the mean value on each individual variable before calculating the sum scores. The variance value (SD) remained the same on this variable even though the missing observations were replaced by the mean value. We argue that it is reasonable to claim that these six items together are able to grasp the degree of involvement in activities that according to Bourdieu [28] the majority of the people in western society regard as highly cultural.

Informal social participation was operationalized with four items in the study. The respondents were asked to report how often during the last week they had done certain activities. Those were: “Paid a visit to someone you know”, “Received a visit”, “Spent more than two hours with friends outside”, and “Went to a café or a meeting place for young people”. The Cronbach’s alpha was 0.69 and in the present study, sum scores were calculated by adding together all individual values and dividing by four. Missing observations on each individual variable were replaced by the mean value before calculating the sum scores. The number of missing observations ranked from 419 to 477. The variance on this variable did not change when the missing observations were replaced by the mean value. In the analyses, the sum score was also aggregated at the municipality level, and thus the individual characteristics of informal social participation worked as proxies for informal social participation at the municipality level. While adjusting for individual social participation in the analysis, we are able to study how social capital as a contextual variable is associated with physical activity, beyond what is due to the association between individual social capital, as a compositional variable, and physical activity. In this way, we are also able to counteract a problem termed the ‘ecological fallacy’ that arises when relationships at the aggregated level are used to infer relationships at the individual level [13].

To assess adolescents’ socioeconomic status (SES), they were asked to comparetheir family’s economic affluence with others. “How are your family’s economic resources compared to everyone else’s?” The response alternatives were: “More affluent than all the others”, “Less affluent than all the others”, and “Same as all the others”. A home affluence scale based on material markers (for example, “does your family own a car?”) is seen a sa useful alternative in assessing family SES, because the adolescent will almost certainly know the answer [29]. In this case, it is hoped that a question about economic affluence in general will reveal both the amount of material resources in the family and the adolescent’s view of the potential for getting what the family wants of material markers. In the analyses, there were two variables: First, “less affluent than all the others” [1], and “same as everyone else in the reference category” (0). Second, “more affluent than all the others” was given the value 1, and “same as everyone else” was the reference category. A subjective measure of the economic situation in the family has also been used in another Norwegian study among adolescents [29]. Other measures of SES such as parents’ income, occupation, and education have resulted in high levels of missing or invalid data. There may be bias in the missing data with higher levels of non-response among children from socioeconomically deprived backgrounds, where issues of employment or education are discussed less [43].

The multilevel nature of the theory of social capital, where social capital at both the individual and community level might be associated with physical activity, in addition to the use of an area deprivation index, makes it necessary to use a multilevel model. In multilevel analyses, the individuals comprised the first level, nested within municipality level. In this study, we expect a statistical dependency between the individuals because we have a hierarchical data set. The objective area deprivation variable and the informal social capital variable at the municipality level operate on all members of the municipality to the same extent. The analyses were run by multilevel linear regression analysis with a stepwise introducing of the group of individual variables in model 1, informal social capital at the municipality level in model 2, deprivation index in model 3, and all variables at both levels in model 4. All analyses were performed in SPSS, version 20.

## Results

Table 1 shows the distribution of the dependent variable: leisure physical activity, and the independent variables informal social participation, cultural participation, and family economic affluence at the individual level on the municipality level. We categorized the 24 municipalities into three groups based on their score in the municipality deprivation index; eight most affluent, eight intermediate municipalities, and eight most deprived municipalities. Concerning leisure physical activity, the average level of physical activity among adolescents living in the eight most affluent municipalities was 3.79 (SD = 2.36), while the average level among those living in the most deprived municipalities was 3.39 (2.28). Concerning informal social participation, the mean value among adolescents was 2.39 (.62) and 2.33 (.58) respectively in the eight most affluent municipalities and in the eight most deprived municipalities. The variable cultural participation had the following distribution: In the most affluent municipalities, the mean value was 1.20 (.30), and in the most deprived municipalities, 1.21 (.30). In the most affluent municipalities, 17.47% answered that their own family is more affluent than all the others, compared with 17.1% and 13.7%, respectively, in the intermediate municipalities and in the most deprived municipalities.

**Table 1:** Distribution of the population, dependent and independent variables by the municipality deprivation index

	**8 most affluent municipalities**		**8 intermediate municipalities**		**8 most deprived municipalities**		**Total**	
	**N = 5940 73.2%**		**N = 1510 18.6%**		**N = 664 8.2%**		**N = 8114 100%**	
	**Mean**	**(SD)**	**Mean**	**(SD)**	**Mean**	**(SD)**	**Mean**	**(SD)**
Leisure physical activity, Dependent variable	3.79	(2.36)	3.67	(2.33)	3.39	(2.28)	3.73	(2.35)
Distribution of independent variables at baseline								
Informal social participation	2.39	(.62)	2.40	(.63)	2.33	(.58)	2.38	(.62)
Cultural participation	1.20	(.30)	1.18	(.28)	1.21	(.30)	1.20	(.30)
	N	%	N	%	N	%	N	%
Family economic affluence- less affluent than all the others	512	8.61	125	8.28	63	9.50	700	8.63
Family economic affluence – same as everyone else	4390	73.91	1148	76.03	508	76.51	6046	74.51
Family economic affluence- more affluent than all the others	1038	17.47	237	15.70	93	14.00	1368	16.86

Mean value and standard deviation (SD), and percentage.

Table 2 shows the distribution of the dependent variable and the independent variables at the individual level on the category variable. Here, 24 municipalities are categorized into three groups according to their value on the variable informal social participation, aggregated on the municipality level. In the eight municipalities classified with the highest level of informal social participation, the mean value for physical activity among the adolescents was 3.81 (2.37). In the eight municipalities classified as having the lowest level of informal social participation, the mean value for physical activity was 3.28 (2.27). Concerning informal social participation at the individual level, the mean value among those living in the municipalities with the lowest level of aggregated social participation was 2.20 (.59) and somewhat higher in the other groups of municipalities, 2.42 (.61) and 2.37 (1.2). In the eight municipalities with the lowest level of informal social participation, we found the highest mean value on the cultural participation variable: 1.23 (.25), and the lowest in the intermediate group of municipalities: 1.18 (.28). Among those living in the municipalities with the highest informal social participation, 9.26% answered that their family was less affluent than all the others, while 7.55% gave this answer in the intermediate group.

**Table 2:** Distribution of the population, dependent and independent variables by informal social participation at the municipality level

	**8 municipalities with the highest level of informal social participation**		**8 municipalities with intermediate level of informal social participation**		**8 municipalities with the lowest level of informal social participation**		**Total**	
	**N = 3962 48.8%**		**N = 3456 42.6%**		**N = 696 8.6%**		**N = 8114 100%**	
	**Mean**	**(SD)**	**Mean**	**(SD)**	**Mean**	**(SD)**	**Mean**	**(SD)**
Leisure physical activity, Dependent variable	3.81	2.37	3.74	2.33	3.28	2.27	3.73	2.35
Distribution of independent variables at baseline								
Informal social participation	2.42	(.61)	2.37	(.62)	2.20	(.59)	2.38	.62
Cultural participation	1.20	.32	1.18	.28	1.23	.25	1.20	.30
	N	%	N	%	N	%	N	%
Family economic affluence- less affluent than all the others	367	9.26	261	7.55	72	10.35	700	8.63
Family economic affluence- same as everyone else	2910	73.45	2607	75.43	529	76.00	6046	74.51
Family economic affluence- more affluent than all the others	685	17.29	588	17.01	95	13.65	1368	16.86

Mean value and standard deviation (SD), and percentage.

In the multilevel analyses, the aim was to see how the level of physical activity among the adolescents was associated with the degree of area deprivation at the municipality level. Other important aims were to see how physical activity is associated with individual variables such as informal social participation, cultural participation and family economic affluence, and the degree of informal social participation at the municipality level. Table 3 reports the unstandardized coefficients and VPS (Variance Partition Coefficient) from the models of leisure physical activity regressed on explanatory variables. In the null model, slightly above 96% of the variation in leisure physical activity was at the individual level and slightly below 4% of the variation at the municipality level. Adjusting for individual variables, age, sex, and family affluence, cultural participation and informal social participation (model 1) led to a decrease in the variance at the individual level from 96.21 to 96.04. These variables thus explain some of the variance in leisure physical activity among adolescents. The variance in the average level of physical activity between the municipalities increased, indicating that the variance in the average level of leisure physical activity between the municipalities in fact was higher when we adjusted for variables at the individual level associated with physical activity.

**Table 3:** The predictors of participation in physical activity

			**Model 0**	**Model 1**	**Model 2**	**Model 3**	**Model 4**
Fixed effects							
Individual level (intercept)			3.500	4.189	-.334	4.361	.570
	Age			-.078**	-.082**	-.081	-.079**
	Sex (male)			.369**	.379**	.379**	.368
	Family affluence economy-						
		More affluent than all the others		.336**	.335**	.339**	.335**
		Less affluent than all the others		-.420**	-.420**	-.428**	-.420**
	Behaviour						
		Cultural participation		-.088**	-.088**	-.090**	-.088
		Informal social participation		.100**	.099**	.102**	.099**
Municipality							
		Informal social participation			.484*		.401#
		Deprivation index				-.043#	-.033
Random effects							
Variance components							
	Municipality level		.213	.217	.175	.192	.150
	VPC (%)		3.79	3.96	3.22	3.44	2.78
	Individual level		5.411	5.253	5.253	5.367	5.253
	VPC (%)		96.21	96.04	96.78	96.55	97.22

Valid N (listwise): 8114.

** = p<0.01, * = p<0.05, # = p<0.10.

In model 1, we see that an increase in age is associated with a decrease in participation in physical activity, and men are more engaged in leisure physical activity than women. We see that the family background “more affluent than all the others” (self-report), is associated with more leisure physical activity, while “less affluent than all the others” (self-report) is associated with less leisure physical activity. These associations were significant at 0.01 levels. Participation in cultural activities was associated with less physical activity, while informal social participation was associated with an increase in physical activity. These were also significant at 0.01 levels.

In models 2 and 3, the variables at level 2 were introduced. In these cases, we do not expect to find any reduction in the variance in physical activity at the individual level. When informal social participation at the municipality level was introduced, the unexplained variance at the municipality level was reduced from .217 to .175, indicating that this variable is responsible for some of the variance in the differences in the mean between the municipalities. Concerning the relative variance, it was reduced from 3.96% to 3.22% at the municipality level in model 1, while it increased from slightly above 96% to 96.78% at the individual level. Municipalities with a high level of informal social participation are associated with an increase in the average level of physical activity. The variable measuring informal social participation has a significant coefficient at p<.05 in model 2, and the variance in the dependent variable was reduced from .217 on model 1 to .175 on model 2. When only area economic deprivation was included in the analyses of model 3, while we adjusted for individual variables, the coefficient was not significant, and the variance in the dependent variable was only reduced from .217 to .192. When both municipality variables were included in the analysis, none of them have significant effects at the five per cent level, but informal social participation has a coefficient with p<.10 at the municipality level. According to Strabec [44], associations with significance values below 0.1 should be mentioned. Taking that into account, these analyses tell us that informal social capital has more effect on level of physical activity than economic deprivation. However, when we include both informal social participation and economic deprivation, the deprivation variable fails to explain the level of average physical activity at the municipality level.

## Discussion

From this study, we can suggest that living in a municipality with less informal social participation at the community level is associated with less physical activity on average among adolescents, when we adjust for individual variables. When we do not adjust for informal social capital at the municipality level, the area deprivation variable is a significant contributor to the variance in the dependent variable at municipality level. From this study, it is plausible to state that social capital at the municipality level seems to be more important than area economic deprivation in explaining variance in physical activity at the municipality level.

These findings tell us that area economic deprivation is not strongly related to the level of physical activity among adolescents, when we adjust for individual variables and informal social capital at municipality level. In accordance with Piro et al. [12], we find that adolescents living in deprivation areas are less physically active. However, this association disappears when we adjust for informal social contact. That is in accordance with Macintyre [13], who claims that collective social functioning and social practices are mechanisms that are relevant in explaining spatial variations in health more so than variations due to individual characteristics or material characteristics of the place of residence. However, we should not simply ignore area deprivation as an important variable. Even though it is less important in the statistical analyses when we adjust for social capital, informal social contact at the municipality level might work as a mediating variable in the association between area economic deprivation and physical activity. An area with a relatively high degree of deprivation might lead to less social capital at the area level, and less social capital in turn leads to a lower level of physical activity. For this reason, area deprivation might still be important.

However, the public sector in Norwegian municipalities may function as a buffer between area deprivation and individual participation in social and physical activities. Tax revenues of Norwegian municipalities are only partly dependent on the income level of the local inhabitants because there is a state-financed redistribution system of municipality incomes at the national level, which according to Lien and Pettersen [45],is a unique collective welfare arrangement. Hence area deprivation only to a limited degree influences the availability of sport parks, and other recreation facilities. The 23 municipalities in North-Trøndelag have 5800 inhabitants on average, and in each municipality there are community centres with gyms, handball halls, swimming pools, and ski parks funded by the profits of betting and generous national government budgets to support local cultural and sports activities. The political purpose of equalization of municipal revenues is to drive social and economic cohesion, as shown by Aarberge et al. [46]. Negative social capital, such as involvement in gangs that may have an adverse influence on social norms for health behavior, is hardly a significant factor in North-Trøndelag, as this county has among the lowest crime rates in the country when it comes to this age group [47].

We find that participation in cultural activities is negatively related to participation in physical activity, while informal social participation is positively related to healthy behaviour such as physical activity. In everyday life, it is plausible to propose that adolescents need to prioritize their activities because of their limited time resources. When they are engaged in activities defined as cultural participation, there seems to be less time to participate in physical activity. Another explanation might be that adolescents with cultural interests do not participate in physical activity because they do not regard themselves as good enough. Instead, they choose tospend time pursuing other avenues and cultivating other interests as compensation. This negative correlation between cultural capital and physical participation might also depend on the measures of cultural capital used in this study, where other cultural activities might be positively related to physical activity. Cultural capital, defined by parents’ level of education, is found to be positively related to physical activity among Norwegian adolescents. Thus, even though participation in cultural activities among the adolescents is negatively related to physical activity, they might still inherit the cultural resources and knowledge about physical activity, which might be important for practising healthy behaviour such as physical activity [27]. An interesting question is the degree to which children and adolescents with a high level of cultural activity and a lower level of physical activity will continue to exclude themselves from physical activity, or will adopt a more physical lifestyle later in life. Informal social participation was positively associated with physical activity, which is in accordance with the findings of Seippel et al. [29]. They found that physical activity among Norwegian adolescents was positively associated with both informal and formal contact with friends, and thus anticipated that those who are physically active have a stronger social position than those who are physically inactive. This fact also provides support to our social capital hypothesis on adolescent physical activity.

In this article, an obvious explanation might also partly be that informal social participation as measured in this study in fact is participation in informal physical activity with friends, and engagement in physical activity is a way of being social with friends. According to Coleman [33], most forms of social capital are created or destroyed as by-products of other activities. It is thus plausible that adolescents who choose to spend time in physical activity also increase their social capital and prestige in their peer group. A causal association, where physical activity predicts later social position, was found in a study among Norwegian fourth graders, whose participation in physical activity and motor functioning at early school age predicted later social standing among class peers [48]. The health-giving and aesthetic functions related to sport are combined with social functions and they become a technique of sociability, reinforcing each other [31].

Studies with such cross-sectional design may be subject to the risk of self-selection, even with adjustment for individual variables as in this study. The reason is that people might be selected in residential areas based on individual attributes that are related to physical activity, which are not included as individual level variables. There might still be other individual variables omitted in this study that can explain variance in physical activity and thus reduce the portion of variation in physical activity given a contextual explanation.

According to Macintyre [13], the differences between compositional and contextual mechanisms are probably more apparent than real. She proposes that individual characteristics of those living in a geographical area might be intervening variables between individuals’ level of physical activity and place of residence. Even though we find that family affluence, as an individual characteristic, is associated with physical activity, family affluence might be dependent on the potential in the place of residence for a family to increase its economic affluence. The potential might be regulated by factors such as distance to relevant workplaces, price levels, and other financial commitments related to place of residence.

Conversely, apparently contextual explanations are nothing other than analyses where individual variables are omitted in the study. Our area, municipality, might be too broad and we might find larger variations between areas in the case of physical activity if we had smaller area units, for example ward level. Even though the Cronbach’s alpha value of the area indicators at the municipality level was high enough statistically, it is difficult to know whether and to what degree the indicators within each of the municipality variables tap into the same aspects and are relatively consistently associated with physical activity. Even though we find significant area effects, this might be dependent on how area exposure was measured, in terms of which exposures were involved, and the spatial level [36]. It is also a limitation when we are using readily available spatial units such as municipality, seemingly independent area units, and ignoring spatial associations between areas. Administrative or spatial units such as municipality might lack an intrinsic meaning in relation to health [38].

## Conclusion

The level of physical activity among adolescents in Norway varies according to place of residence. When we adjust for individual variables and social participation at the municipality level, area deprivation is not able to explain the geographical variance in the average level of physical activity between the municipalities in Nord-Trøndelag County. What we do find is that the amount of informal social contact between adolescents in a municipality explains some of the variance at the municipality level, when we adjust for the same variable at the individual level. Finally, we find that adolescents who participate in cultural activity such as theatre, shows, film/photo, concerts, choirs, libraries, and cinema participate in physical activity to a lesser extent. Those who are socially active with their friends, informally, are more physically active in their spare time than those who participate less in informal social activities with their friends. This might mean that participation in physical activity for some adolescents is a way of being informal with their friends, and physical activity in fact is social participation. In further research, we need to be aware of this issue, where healthy behaviour, in this case physical activity, might be the same as social participation. Despite these shortcomings, it is reasonable to state that the amount of social capital, both at the individual and community level are strengthening the individual’s ability to take part in physical activity.

## Competing interests

The authors declare that they have no competing interests.

## Authors’ contributions

BL planned the study design and drafted the manuscripts. AB carried out the statistical analyses. RA participated in drafting the manuscript and in planning of the study. All authors read and approved the final manuscript.
